# Preimplantation genetic testing for recurrent autosomal dominant osteogenesis imperfecta associated with paternal gonosomal mosaicism

**DOI:** 10.3389/fgene.2022.1011833

**Published:** 2022-10-05

**Authors:** Haiyan Bai, Xiaofang Li, Xitong Liu, Wenhao Shi, Bin He, Ruiyang Wei, Juanzi Shi

**Affiliations:** ^1^ The ART Center, Northwest Women’s and Children’s Hospital, Xi’an, China; ^2^ Genetic Medical Center, Xi’an, Jiangsu, China; ^3^ Yikon Genomics Company, Ltd., Suzhou, China

**Keywords:** preimplantation genetic testing for monogenic disorders, gonosomal mosaicism, osteogenesis imperfecta, sequencing, single nucleotide polymorphism

## Abstract

**Research Question:** How to prevent the transfer of a mutation causing osteogenesis imperfecta (OI) to offspring in a couple with recurrent adverse pregnancy outcomes, when the male partner is a gonosomal mosaic carrier.

**Design:** High-throughput sequencing and first-generation DNA sequencing were performed using the tissues from an aborted fetus and its parents. Regions 2 Mb upstream and downstream of the COL1A1 gene were subjected to multiplex PCR to identify single nucleotide polymorphisms (SNPs) and family haplotypes associated with the disease-causing mutation. Single-cell whole-genome amplification and sequencing were performed on trophoblasts cultured *in vitro* for 5–6 days to construct embryonic SNP haplotypes, and first-generation sequencing was used for pathogenic locus verification and aneuploidy screening. Preimplantation genetic testing for monogenic disorders (PGT-M) was also performed.

**Results:** The aborted fetus was heterozygous for the COL1A1 mutation c.1454G>A (chr17-48272089, p.Gly485Asp) suspected to cause OI. The variant was also detected in the peripheral blood cells and sperm of the male partner, who appeared to be a gonosomal mosaic carrier of the mutation. Three morphologically usable blastocysts were obtained *in vitro* and successfully expanded after a trophectoderm biopsy. Two blastocysts were unusable owing to aneuploidy; however, one was euploid and did not carry the paternal mutation. Post-transfer gestation was confirmed by systematic B-scan ultrasound, and amniocentesis findings were consistent with the PGT-M results.

**Conclusion:** Parental gonadal mosaicism was the cause of recurrent terminated pregnancies due to fetal skeletal dysplasia. Using PGT-M to select embryos without the paternal pathogenic mutation prevented the vertical transmission of OI in this family, and a successful pregnancy was achieved.

## Introduction

Osteogenesis imperfecta (OI), also known as brittle bone disease, is one of the most common monogenic disorders with a prevalence of 1 in 15,000–20,000 neonates. OI is characterised by low bone mass, increased bone brittleness, and recurrent fractures due to mutations in the genes encoding type 1 collagen, a major bone matrix protein, and/or those associated with collagen metabolism (Marini and Forlino, 2017). To date, 21 OI causative genes have been reported. Type 1 collagen comprises three chains, two α1 (COL1A1) and one α2 (COL1A2), arranged in an ordered triple helix structure. Mutations in the COL1A1 and COL1A2 genes are the principal cause of OI and they have an autosomal dominant inheritance pattern (Sato et al., 2016).

As there is no effective treatment for OI, preimplantation genetic testing for monogenic disorders (PGT-M) is conducted for families carrying causative mutations. This is the best strategy to avoid the physical and psychological trauma of pregnancy termination when this mutation is identified during prenatal testing.

With autosomal dominant monogenic disorders, offspring typically have disease-associated *de novo* mutations (DNMs) if both parents have normal phenotypes and genotypes. However, a possibility of gonadal mosaicism has been suggested for some families, which have consecutive offspring with the same abnormal phenotype (Altarescu et al., 2012). In the present case, there was no typical OI phenotype in either parent; nevertheless, B-scan ultrasounds in all three pregnancies indicated intrauterine fetal OI. Genetic analysis revealed a pathogenic COL1A1 mutation in the aborted foetal tissue and the male partner, who appeared to have gonosomal mosaicism. After genetic counselling, PGT-M was performed, and it successfully prevented the vertical transmission of the OI-associated genetic defect from the male parent to the offspring.

## Materials and methods

### Ethical approval

This study involving human participants was reviewed and approved by Northwest Women’s and Children’s Hospital, Xi’an, China. The patients/participants all provided written informed consent to participate in this study. Written informed consent was obtained from the authors and participating patients for the publication of any potentially identifiable images or data included in this article.

### Clinical data

The 35-year-old female partner complained of irregular menstruation 5–7/50 days of a moderate volume and dysmenorrhea (+) but had no abnormalities on physical examination. The 37-year-old male partner was 170 cm and had partial curvature of the fourth and fifth fingers in both hands, which he could not bring together; he also could not join his knees. He had progressive hearing loss in the left ear since early adulthood (age 22), a history of chronic enteritis for 5 years, and a high rate of morphologically abnormal sperm.

This couple had three pregnancies, which were terminated at 18 weeks of gestation because of abnormal B-scan ultrasound results. In 2010, ultrasonography suggested dysplasia of the fetal zygomatic bone, and in 2013, the long limb bones of the fetus were found to have a “dumbbell”-like morphology, and the two thighs and the forearms formed O-shapes, suggesting OI. The same diagnosis was made in 2018, when the fetus showed a thin cranial plate; narrowed thorax; shortened long bones; malformed joints; unclear tibia, fibula, ulna, and radius in the limbs; and the formation of angles in some areas.

### Detection of disease-causing gene loci

Genetic testing was performed using high-throughput sequencing of genomic DNA extracted from tissues obtained from the third aborted fetus and the median cubital veins of each parent. Disease-causing loci were verified using first-generation sequencing of the genomic DNA from the amniotic fluid sample of the third pregnancy, blood samples from both partners, and semen sample from the male partner, which were collected using conventional means (Zong and Lu, 2012). Database and literature surveys and conservation analysis for mutation site were performed, and 3D protein structures were assembled.

### Family pre-testing

In pre-testing, high frequency SNP loci within the causal gene and the 2 Mb upstream and downstream regions were screened as genetic markers using specific primers designed online (http://www.ampliseq.com). Haplotypes were constructed based on SNP locus analysis. Capture amplification, purification, and library construction were performed using parental and fetal DNA to identify haplotypes associated with the disease-causing mutations and to establish a foundation for subsequent embryonic SNP linkage analysis.

### PGT-M-assisted reproductive technology

PGT-M was initiated after successful pre-testing. Eight eggs were obtained using the follicular-phase long protocol, including six MII eggs fertilised by intracytoplasmic injection of a single sperm, and cultured in sequential media until day 6. Trophectoderm biopsy and genetic testing were performed on three morphologically usable blastocysts, which were then frozen by vitrification. All these can refer to previous study for details (Yan et al., 2015).

### Single-cell whole-genome amplification and sequencing

Trophectoderm biopsies of the blastocysts and WGA were performed using a universal sample processing kit for gene sequencing (ChromSwiftTM, XK-028, Yikon Genomics) according to the manufacturer’s instructions. To analyse the embryonic chromosomal set for euploidy, WGA products were fragmented for library construction and sequenced using the Illumina Nextseq 550 system. In addition, SNP haplotype analysis of the WGA products and first-generation sequencing of mutation loci were performed to determine the genotype of the embryos. Based on these analyses, euploid embryos without disease-causing mutations or any other abnormalities were selected and recommended for clinical transfer.

### Embryo transfer and follow-up

Human chorionic gonadotropin (hCG) levels were measured in the plasma of the female partner at day 12 after embryo transfer, and a B-scan ultrasound was performed at day 28 to confirm clinical pregnancy. At 20 weeks of gestation, fetal systemic B-scan ultrasound and amniocentesis were performed to verify fetal chromosomal euploidy and the genotype.

## Results

### Genetic testing results and analysis

**FIGURE 1:**
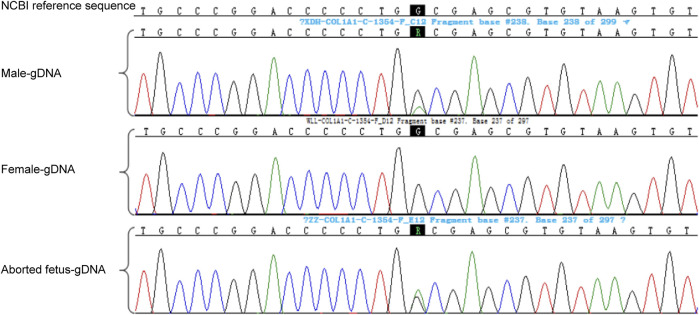
Validation of the mutation locus in the family.

**FIGURE 2:**
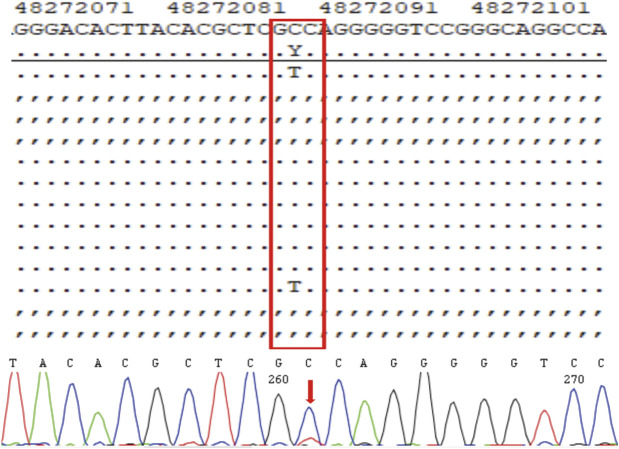
High-throughput sequencing results and electropherogram of first-generation DNA sequencing for male peripheral blood cells.

Genetic testing of the aborted fetal tissue *via* high-throughput sequencing suggested a heterozygous mutation in the COL1A1 gene: c.1454G>A, chr17-48272089, p.Gly485Asp. The verification of the mutation in the parents indicated that it originated from the male partner (Figure 1). Second-generation sequencing analysis revealed that the percentage of mutations in the peripheral blood specimen from the male partner was 15.1% (73:13, wild type to mutant read number ratio). First-generation sequencing also revealed the presence of a small heterozygous peak at this locus in the male partner, indicating that his percentage of somatic mosaicism in the identified locus was low (Figure 2). Considering the history of the three adverse pregnancy outcomes, gonosomal mosaicism in the male partner was suspected.

**FIGURE 3:**
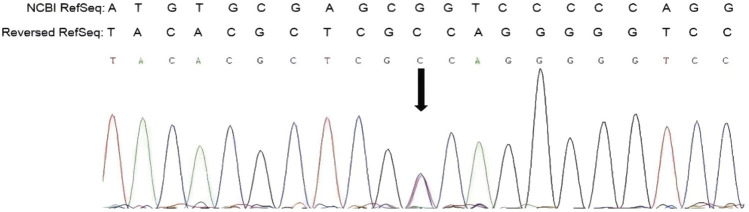
First-generation DNA sequencing of male partner’s sperm.

**FIGURE 4:**
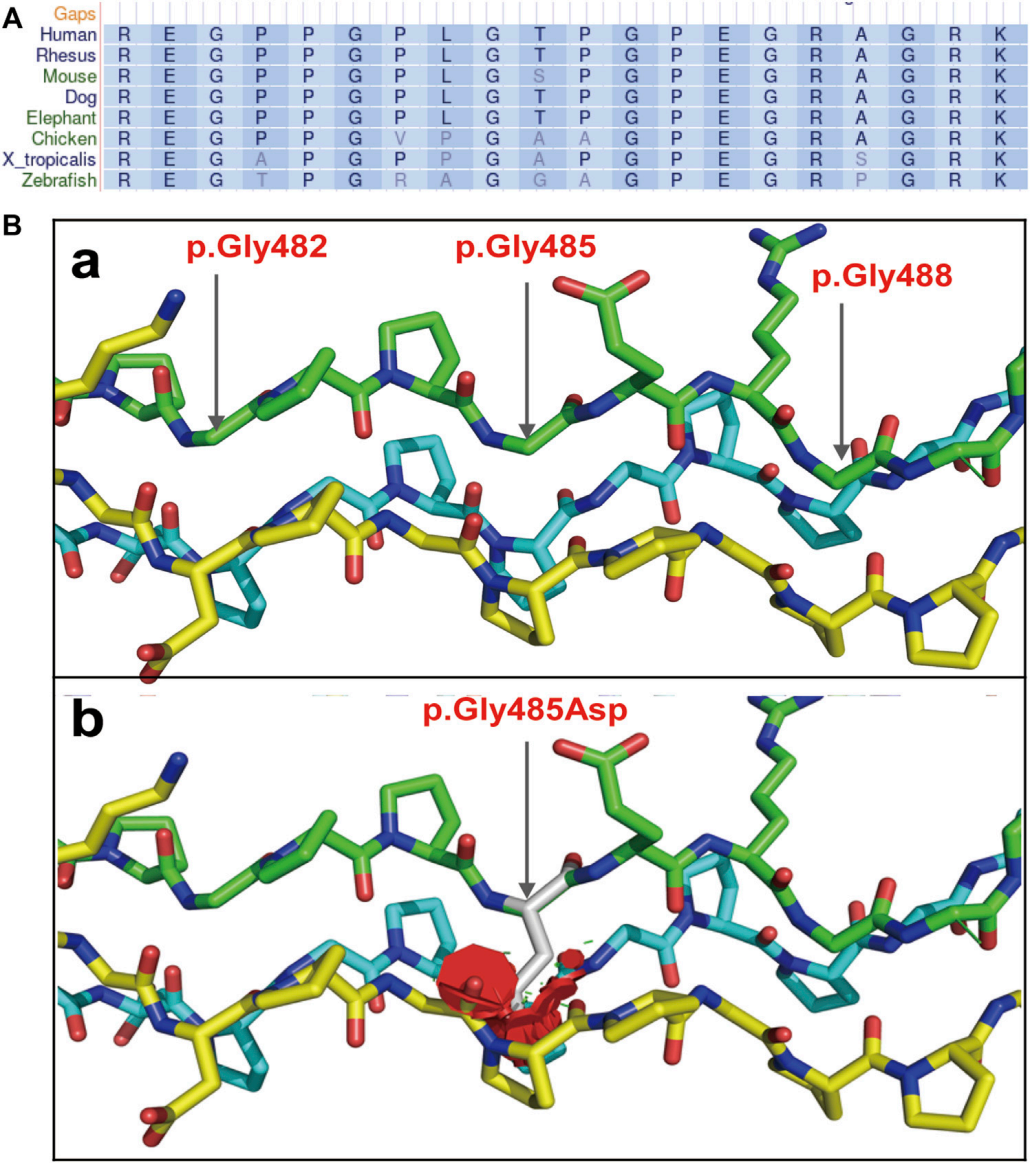
Collagen secondary and tertiary structures. **(A)** Gly485 is highly conserved among collagens of multiple species. **(B)** 3D structure of collagen regions around the mutation site: (a) the normal collagen spatial structure; (b) the changed spatial structure due to p.Gly485Asp mutation.

The COL1A1 c.1454G>A mutation was detected in the sperm, which confirmed gonosomal mosaicism in the male partner (Figure 3). Database and literature surveys for c.1454G>A p.Gly485Asp Exon21 (c.1354_1461) and mutation site conservation analysis were performed, and 3D protein structures were assembled (Figures 4A,B). The results showed that the glycine residue at position 485 was highly conserved in several species (Figure 4A) and that the p.Gly485Asp mutation resulted in a change in the normal collagen spatial structure because of the repulsion between Asp485 and Gly residues 482 and 488 (Figure 4B). The pathogenicity level of the p.Gly485Asp mutation was classified as “possibly pathogenic.”

**FIGURE 5:**
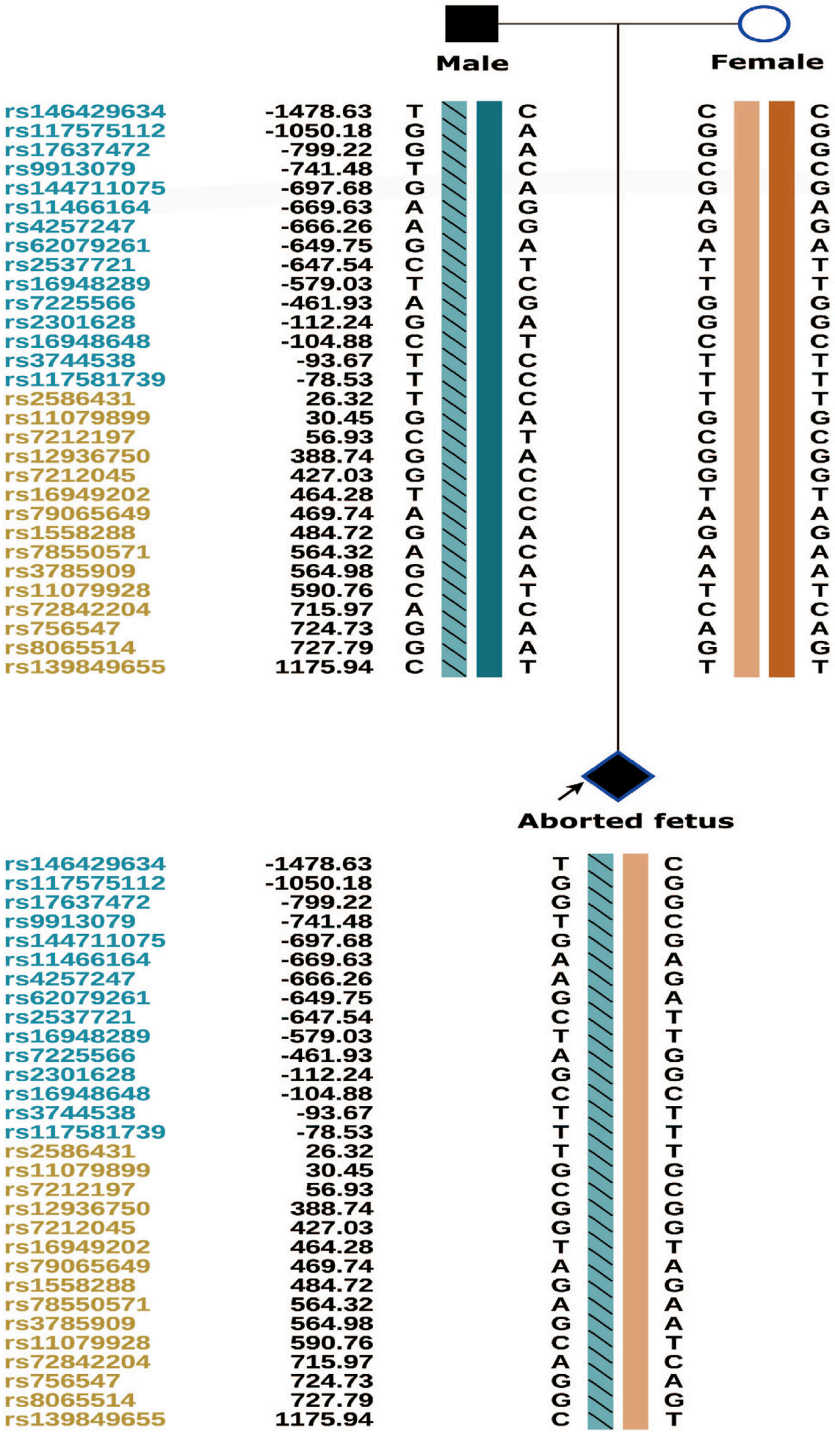
Results of family pre-testing SNP linkage analysis.

### PGT-M testing results and pregnancy outcome

The couple was offered genetic counselling and informed about OI, the treatment approaches, the life that a child with OI can expect, and the risk of mutation recurrence in the offspring by rare disease experts. The couple requested PGT-M for assisted reproductive technologies. The results of PGT-M for SNP haplotype construction were consistent with those of the genetic testing, and the haplotype associated with the disease-causing mutation was confirmed to have originated from the male partner (Figure 5).

**FIGURE 6:**
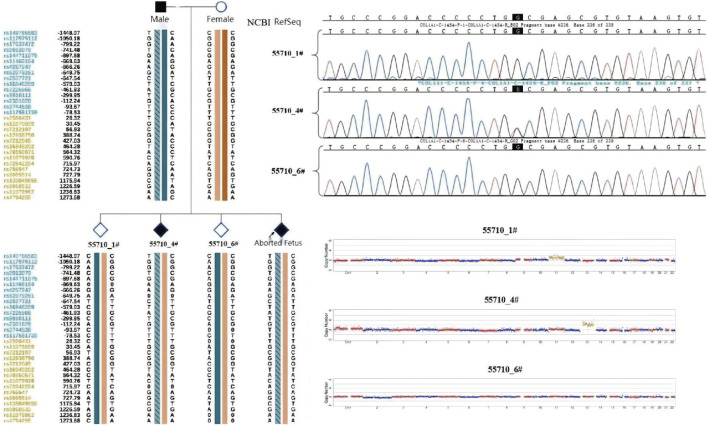
Embryo testing results (including haplotype analysiss, sanger sequencing for mutation locus testing and CNV testing, revealed that two of the blastocysts were aneuploid, whereas one was euploid and did not carry the paternal mutation).

**FIGURE 7:**
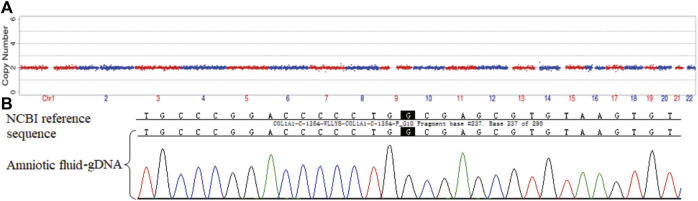
Prenatal diagnosis by amniocentesis at 20 weeks of gestation. CNV **(A)** and genetic testing **(B)** results for amniotic fluid-derived cells.

The three morphologically usable blastocysts were each successfully expanded after a trophectoderm biopsy. Embryo testing revealed that two of the blastocysts were aneuploid, whereas one was euploid and did not carry the paternal mutation (Figure 6). After genetic counselling, the female partner underwent frozen-thawed embryo transfer with blastocyst 55710_6# in May 2021. The blood hCG level was 455.96 mIU/ml at day 12 after transfer, and the B-scan ultrasound at day 28 indicated the survival of a singleton intrauterine fetus. Prenatal diagnosis by amniocentesis at 20 weeks of gestation indicated a normal karyotype, no abnormal copy number variations (CNVs), and no COL1A1 c.1454G>A, chr17-48272089 mutation (Figures 7A,B). Maternal examination results were normal, and no abnormalities were identified in the fetal systemic B-scan ultrasound. A healthy male neonate weighing 3,450 g was delivered by caesarean section at 39+1 weeks of gestation.

## Discussion

PGT-M is now widely used for detecting monogenic disorders before embryo implantation. The goal is to genetically diagnose embryos prior to pregnancy to avoid the implantation of embryos that are genetically high-risk, thereby improving pregnancy outcomes (Richards et al., 2015). OI is one of the most common monogenic disorders for which PGT-M is utilised (Sillence et al., 1979).

In this case, the parents did not have a typical OI phenotype; only the male partner had partial curvature of the finger bones and unilateral hearing loss in adulthood. A B-scan ultrasound revealed intrauterine fetal OI in three terminated pregnancies, and genetic testing of the last aborted fetus revealed that it was heterozygous for the COL1A1 c.1454G>A mutation. The verification of the mutation in the parents showed that its percentage in the peripheral blood of the male partner was 15.1%, suggesting somatic mosaicism. This diagnosis would explain mild phenotype and indicates a paternal origin for the mutation in the fetus. Considering the history of three adverse pregnancy outcomes and results of the sperm analysis, gonosomal mosaicism was confirmed in the male partner, and PGT-M was suggested for assisted reproduction. The phenotype of the male partner was atypical, and that of the proband was inferred from the intrauterine B-scan ultrasound examination, which provided evidence that the co-segregation of the phenotype and genetic variant was weak, and caution was thus taken to confirm the pathogenicity of the mutant locus prior to PGT-M.

While the COL1A1 gene is associated with OI, the mutation c.1454G>A p.Gly485Asp Exon21 (c.1354_1461) has not been previously reported. Database and literature surveys were performed in accordance with the American College of Medical Genetics and Genomics (ACMG) classification and criteria for mutations (Richards et al., 2015). The analysis of gene conservation and splice-site impacts suggested that the mutation could have deleterious effects on the gene or gene product (PP3 class) but was not found in normal control populations in the database (PM2 class). However, considering that another mutation affecting the same amino acid position (c.1454G>C, p.Gly484Ala) is evidently pathogenic and that the Gly→Asp substitution may affect the collagen structure more significantly than the Gly→Ala, it was suspected that p.Gly485Asp could lead to a more severe phenotype (PM5 class). Amino acid changes in Gly-X-Y repeats of the collagen triple helix (residues 407–476) affect the functional region of the protein, and multiple mutations detected in the surrounding areas have been identified as pathogenic or likely pathogenic (PM1 class). Thus, mutation c.1454G>A p.Gly485Asp Exon21 (c.1354_1461) could be classified as “possibly pathogenic” (PM1 + PM2 + PM5 + PP3). Missense mutations disrupt the triple helix domain of collagen, altering protein stability and causing defects in severe forms of OI (Zhytnik et al., 2020). The mutation detected in the present case was not found in the Human Exon Database (ExAC), Genome Aggregation Database (gnomAD), Mastermind, ClinVar, Online Mendelian Inheritance in Man (OMIM), or Human Gene Mutation Database (HGMD). Considering the fetal B-scan ultrasound results obtained in 2013 and 2018, it was suggested that the fetuses of this couple may have been affected with severe OI of type II (Maioli et al., 2019).

Recurrent lethal OI is usually caused by dominant mutations associated with parental mosaicism (Chen et al., 2013). An individual containing two or more genetically distinct cell lineages is considered to have somatic, gonadal, or gonosomal mosaicism; the male partner in the present case was classified as the latter type. Gonosomal mosaicism is a type of somatic mosaicism that occurs very early in the development of an organism and is thus present within both germline and somatic cells (Zlotogora, 1998). According to linkage analysis of the aborted fetal tissue carrying c.1454G>A, some percentage of the embryos with the same male partner-derived haplotype did not have this mutation (which is due to gonadal mosaicism in the male). The aim of the PGT-M was to identify mutation-free embryos. However, linkage analysis for point mutations can exclude some normal embryos, producing false positives and resulting in wasted embryos. In addition, the limitation of this method is that it cannot rule out whether the mutation is in two haplotypes, resulting in the inability to identify false negative embryos. Although the probability of that happening is extremely low. Based on the characteristics of single molecule detection and long read sequencing, the third generation sequencing has unique advantages in haplotype analysis. Third-generation sequencing of peripheral blood or sperm DNA from the male partner could be used to directly identify which haplotype the mutation site was located in. Both false negatives and false positives can be avoided. At present, the cost of third-generation sequencing is too high, and it has not been effectively applied in clinical practice. If the cost of third-generation sequencing can be further reduced and accepted by the public, it may be a better choice.

In the present case, genetic testing of the proband foetus fortuitously revealed heterozygosity of the COL1A1 c.1454G>A mutation. Verification using the parents’ peripheral blood cells suggested inheritance from the male partner and led to the successful verification of gonadal mosaicism. In clinical practice, it is more common when the mutation is present in the blood-derived DNA of a proband but is absent in that of both parents. Such cases are typically defined as a DNM, which is considered to arise in a single sperm or egg during meiosis and thus having a very low theoretical probability of recurrence. However, gonadal mosaicism can increase the recurrence risk of the same mutation, as is evident in the present case. In these families, a parent appears phenotypically normal but has multiple children affected with a penetrant, autosomal dominant or X-linked disorder. Such a pedigree can be explained by germline mosaicism in which a proportion of germ cells harbor the damaging allele, which may be transmitted to progeny (Thorpe et al., 2020). Some disorders are particularly prone to germline mosaicism and OI is one of them (Pyott et al., 2011). Gonadal mosaicism may arise from somatic mutations in the parental generation, which are undetectable in peripheral blood but could be present in germ cells; when passed to the offspring, such mutations appear to be DNMs (Morrow, 2020). Therefore, some studies reveal that to identify paternal gonadal mosaicism, the sequencing of sperm DNA is preferable to that of peripheral blood DNA. This suggests that the direct assessment of known pathogenic variants in the sperm and their classification as low- or high-risk for recurrence are important for affected families (Breuss et al., 2020).

Several cases in which parental gonadal mosaicism causing disease in offspring have been identified, primarily through prenatal genetic diagnosis (Pyott et al., 2011; Chen et al., 2013; Zhou et al., 2018). In the present case, the male partner exhibited gonosomal mosaicism, and PGT-M was performed to select embryos without the disease-causing mutation. Single-cell genome amplification and high-throughput sequencing using multiple annealing and looping-based amplification cycles were conducted for blastocyst screening. An embryo free of deleterious mutations of paternal origin was selected for transfer, resulting in the birth of a healthy baby. Thus, vertical transmission of OI was successfully prevented in this family. The present study has broadened the clinical strategy for the birth of healthy offspring from individuals with gonosomal mosaic carrier.

### Key message

In the present case, genetic analysis revealed a pathogenic COL1A1 mutation in the aborted foetal tissue and the male partner, who appeared to have gonosomal mosaicism. After genetic counselling, a successful pregnancy was achieved by performing PGT-M, which prevented vertical transmission of the OI-associated genetic defect in the family.

## Data Availability

The datasets for this article are not publicly available due to concerns regarding participant/patient anonymity. Requests to access the datasets should be directed to the corresponding author.
